# Reliability in Healthcare Simulation Setting: A Definitional Review

**DOI:** 10.7759/cureus.8111

**Published:** 2020-05-14

**Authors:** Sunny J Yauger, Abigail Konopasky, Alexis Battista

**Affiliations:** 1 Healthcare Simulation, Fort Belvoir Community Hospital, Fort Belvoir, USA; 2 Health Professions Education, Uniformed Services University of the Health Sciences, Bethesda, USA

**Keywords:** reliability, definition, attributes, social practice, error, simulated participant, performance, simulation design, replicability, reproducability

## Abstract

The construct of reliability in health professions education serves as a measure of the congruence of interpretations across assessment tools. When used as an assessment strategy, healthcare simulation serves to elicit specific participant behaviors sought by medical educators. In healthcare simulation, reliability often refers to the ability to consistently reproduce a simulation and that reproducing a simulation setting can consistently expose participants to the same conditions, thus achieving simulation reliability. However, some articles have expressed that simulations are vulnerable to error stemming from design conceptualization to implementation, and the impact of social factors when participants interact and engage with others during participation. The purpose of this definitional review is to examine how reliability has been conceptualized and defined in healthcare simulation, and how the attributes of simulations may present challenges for the traditional concept of reliability in health professions education.

Data collection and analysis was approached through a constructivist perspective and grounded theory strategies. Articles between 2009-2019 were filtered applying keywords related to simulation development and design. Data winnowing was structured around a framework viewing simulation as a social practice where participants interact with simulation setting attributes.

Healthcare simulation setting reliability is not directly defined but described as errors introduced by the interactions between simulation design attributes and tasks performed by simulated participants. Based on the ontology of simulation’s design attributes believed to introduce setting errors, lexical terms related to reliability suggest how simulated participants are trained to refine or maintain their performance tasks that aim to mitigate errors.

To achieve reliability in health professions education (HPE) and healthcare simulation, both domains seek to assess the consistency of a construct being measured. In HPE, reliability refers to the consistency of quality measures across a range of psychometric tests used to assess a participant’s medical aptitude. In healthcare simulation setting, reliability refers to the consistency of a simulated participant (SP) performing a task that is tailored to mitigate errors introduced by simulation design attributes. Consequently, inconsistencies in SP performance subject participants to setting errors exposing them to unequal conditions that influence competency achievement.

What is already known on this subject: Performance competency assessment using healthcare simulation is increasingly common. The types of design attributes incorporated into a simulation setting. The use of incorporating simulated participants into a simulation setting. Simulated participants require training prior to simulation setting implementation.

What this paper adds: Identifies attributes of a simulation setting that are most commonly thought to interfere with setting reliability. Identifies the relationships among setting attributes and simulated participant performances that influence setting reliability. Identifies terms tied to the achievement of simulation setting reliability. Examines simulated participant training processes aimed to mitigate errors introduced by simulation design attributes.

## Introduction and background

A concern with healthcare simulation is its vulnerability to error stemming from its implementation flexibility and the impact of context-related factors (e.g., availability of diagnostic tools, simulator delay or troubleshooting) when participants interact and engage in a simulation setting [1]. Adding to this are social interactions between participants (e.g., learners, clinicians) and simulated participants. For instance, variability may arise from a simulated participant's performance. A simulated participant (SP) is an “inclusive term to include all role players in any simulation context” with varying levels of experience or when they are selected from different population pools (e.g., faculty, medical students, children) [2]. These dynamic interactions can subject participants' goal-oriented activities to measurement errors when making critical decisions that influence their academic progression and license.

Viewed as a system, the ecological complexity of a simulation setting may not afford its capture by the traditional concept of reliability, particularly the assumed implications that healthcare simulation settings can be replicated by others [3]. In health professions education (HPE), the construct of reliability traditionally focuses on education outcome measurements (e.g., education achievement), something that does not extend to include the social aspects of a simulation. Thus, a healthcare simulation setting challenges the traditional concept of reliability in HPE, raising an important question in simulation-based education and research: how do we define “simulation reliability”? This study examines how reliability has been defined (explicitly and implicitly) in healthcare simulation and what additional related terms are tied to it. The research process illuminated how simulated participant performance training is operationalized.

The construct of reliability in health professions education and simulation

Reliability plays a central role in education assessment and social science research where “reliable data in HPE provides the foundation for trustworthy evidence needed to inform and enhance effective practices” [1]. This core concept in HPE indicates that the reproduced scores of an individual across numerous repeated measures will cancel out random error [4-7]. This process results in an individual’s “true score.” When an individual’s true score or collective true scores across groups are in agreement, the connections can be translated to a quantifiable measure to estimate reliability. For assessment scores to be interpreted or taken seriously, they must be reproducible; if not, then a meaningful interpretation is questionable [1]. In healthcare simulation, this same idea quantifying the reliability of high-stakes performance tests (e.g., objective structured clinical examinations) helps identify the primary construct being measured (e.g., education achievement) as opposed to random errors generated by contextual factors (e.g., training location and the purpose of the simulation) [8].

In healthcare simulation, in comparison to the HPE assessment literature, reliability has been defined as the “consistency of a simulation activity or the degree to which a simulation activity is measured in the same way each time it is employed under the same conditions with the same participants” [9]. This approach assumes that by implementing the same designed simulation, participants will be exposed to the same conditions, thus achieving a fair and trustworthy experience for each participant to optimally perform their activity driven goals towards competency achievement.

Furthermore, when discussing standardized test conditions, McGaghie and Issenberg indicate that examinees are the only “variable” and that “fixed” conditions pertain to setting attributes [10]. In other words, in HPE more broadly, reliability refers to the consistency and accuracy of the measurement tool, while in the specific case of simulation design, it refers to the consistency and accuracy of the simulation setting developed [11, 12]. Yet, uses of these concepts and terms of simulation reliability do not appear to be fixed across studies; further research is needed to clarify the concept and terminology of reliability when applied to a healthcare simulation setting. Moreover, there is a lack of evidence on how to carry out simulation design and achieve operational reliability for simulation-based education and research [11]. Untangling the lexical differences of “reliability” between HPE and simulation-based education and research may avoid misinterpretations and miscommunications to assist those wishing to design reliable simulation settings.

Designing for reliability in simulation

One of the most common ways the construct of reliability has been discussed in the healthcare simulation literature is with regards to simulation design practices. For example, Munroe et al. contend that “to achieve reliability in simulation, settings must be designed and planned so they may be reproduced consistently to ensure all participants are exposed to the same conditions” [11]. In designing for reliability, healthcare simulation designers often consider simulation attributes. Attributes are often discussed as artifacts that mediate participant activity goals and influence social practices, for example, the scope of simulated participants, medical equipment, simulator modality [13, 14]. Attributes, however, do not exist in isolation. Their properties permeate throughout a setting, interacting with each other and influencing participant interactions as part of a dynamic system [15]. In other words, a simulation can be designed from a standardized perspective, however, as a complex system, the whole setting is not equal to the sum of its parts. The dynamics of a simulation setting lead to activity variance challenging the concept of reliability in healthcare simulation as it is traditionally used in HPE.

The current study

The purpose of this study was to examine how reliability has been defined in healthcare simulation and what additional related terms are tied to it. The proposed research questions were:

1. How has reliability in healthcare simulation has been defined and described in the literature?

2. What other relevant terms are tied to the concept of reliability in the context of a simulation setting?

This research aims to raise awareness and open a debate about how attributes of a simulation setting challenge the traditional concept of reliability in HPE. Defining reliability in healthcare simulation could enhance communication and lead to more effective interactions among global medical educators, healthcare simulation educators, and researchers as they seek to create reliable simulations. Furthermore, these discussions may lead to research on how reliability for a simulation setting is achieved in simulation-based education and research, and provide a foundation for gathering trustworthy evidence in the context of a simulation setting. This is an increasingly common focus for HPE research and scholarship. To the best of our knowledge, this is the first definitional review of reliability in healthcare simulation.

## Review

Study design

**Figure 1:**
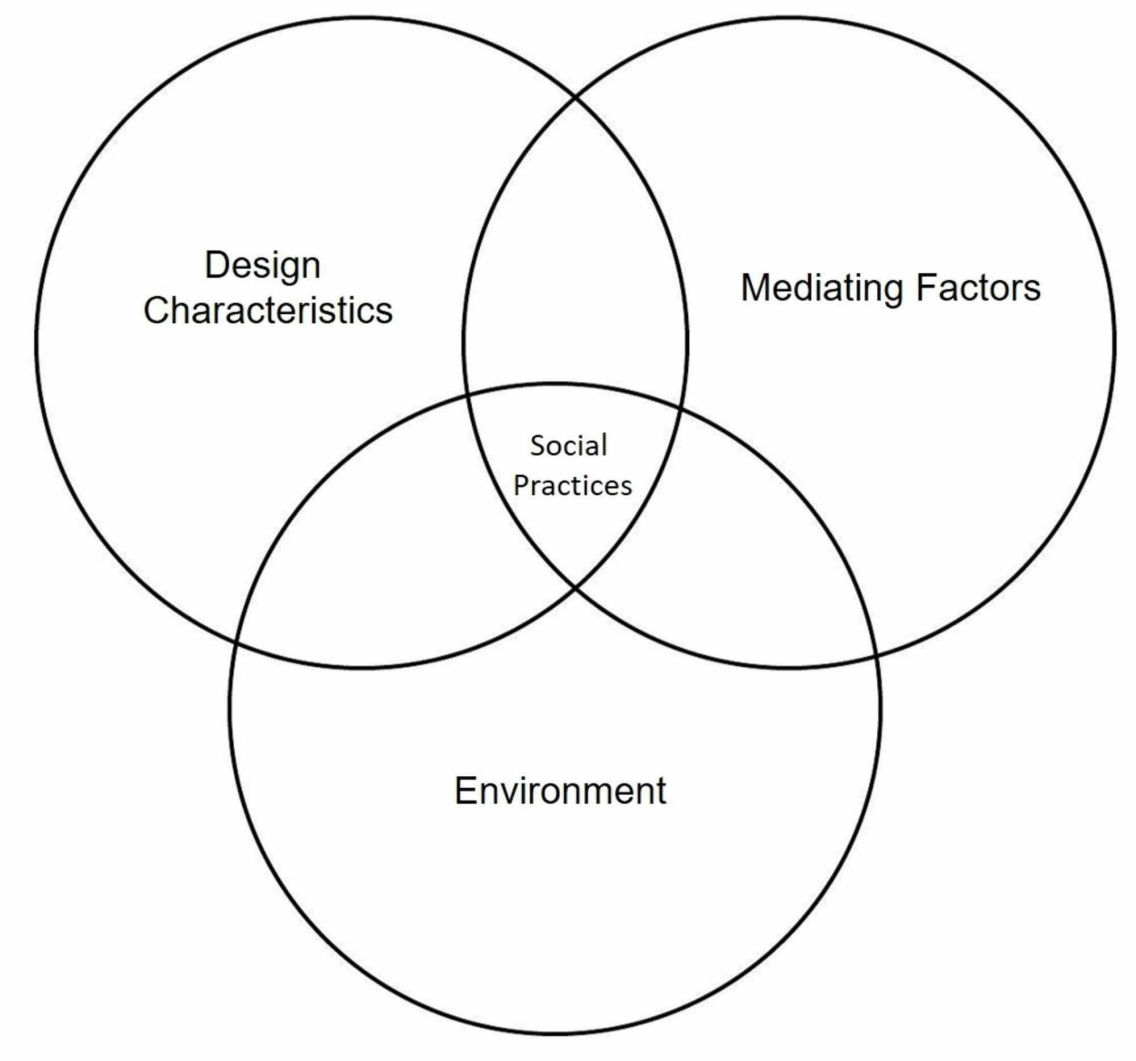
Healthcare simulation setting framework

In this mixed-method definitional literature review, the research was explored through a constructivist paradigm and grounded theory methods. A constructivist paradigm views knowledge as actively constructed and co-created through human interactions and relationships [16]. In this view, healthcare simulation is a social practice where participants interact in a goal-oriented fashion with setting attributes to elicit a behavioral response (Figure 1) [17]. This simulation setting framework served to investigate the concept of healthcare simulation reliability. The application of grounded theory strategies allowed for iterative and constant comparison throughout the data collection process. This strategic combination provided a flexible approach to draw from various perspectives in the literature. The Standards for Reporting Qualitative Research serves as the reporting guidelines [18].

Search strategy

With the assistance of a medical librarian, a search string in PubMed targeted HPE simulation-users (Appendix I). Inclusion criteria were articles published between 2009-2019 regardless of article research design. Keywords related to simulation setting and design included simulation design, scenario design, scenario setting scenario development, simulation development, simulation environment, in-situ simulation, and simulation milieu. Drawing from Norman, the terms agreement, reproducibility, association and correlation were included in the keyword search process [19]. Exclusion criteria included grey literature and articles related to gaming or virtual reality, systematic reviews, and non-English literature. Although virtual reality is a simulation modality, it was excluded on the basis that a virtual environment may engender a different sense of presence [20].

Article selection method

QSR International’s NVivo data analysis software was used to support search processes, data collection efforts, and data management. To initiate exploration of contextual data centered around reliability and its related terms, a stemmed-word query was used to reveal and quantify the most frequent synonyms from each article’s abstract resulting from the initial literature search. Query specifications were set to generate words with a minimum of five characters to omit conjunction and determiner words (e.g., the, a, many, an, but, this). Reliability terms identified were added to a word search query that highlighted individual words throughout the articles for ease of detection. Highlighted words operationalized the open coding cycle for data collection. Open coding “fractures or splits the data into individually coded segments” to use for future reference [21]. During exploration, no new terms were identified.

Data collection included an iterative two-step process that sought to code discrete contextual data related to reliability and collect simulation setting metadata. To organize metadata and map each article’s simulation setting, a spreadsheet was developed containing simulation setting classifications (e.g., simulation modality) and associated attributes (i.e., simulated participant, human patient simulator, task trainer).

Open coding was operationalized followed by axial coding which supports the reassembly of data to seek relationships between categories and sub-categories [22]. This approach uncovered themes and sub-themes related to how healthcare simulation setting reliability is conceptualized and achieved.

The exploration of contextual data and interpretations of an article’s described simulation setting assisted in discerning the ontological difference among reliability when a simulation is employed as an education assessment tool, the subject of clinical research, or as an environment of research [10, 11, 23]. The combination of discerning context and classifying setting attributes winnowed the data, a process of focusing on some of the data and disregarding other parts of it [24].

Techniques to enhance trustworthiness

Collaboration with a medical librarian enhanced search effort to maximize the identification of articles for possible inclusion. Furthermore, weekly meetings were held with two interdisciplinary researchers to discuss findings and assumptions during the data collection and analysis processes. One researcher has expertise within healthcare simulation and has a specialization in simulation-based learning and the conduct of systematic reviews. The second researcher has expertise in qualitative and mixed-methods research design. These meetings provided peer examination of the analysis as recommended by Saldana [21]. Accountability and an audit trail were captured using video recordings of each meeting.

Reflexivity

The first author conducted all literature reviews. Her own healthcare simulation experience has taken place since 2008 until the present in simulation center operations and management.

Article selection

**Figure 2:**
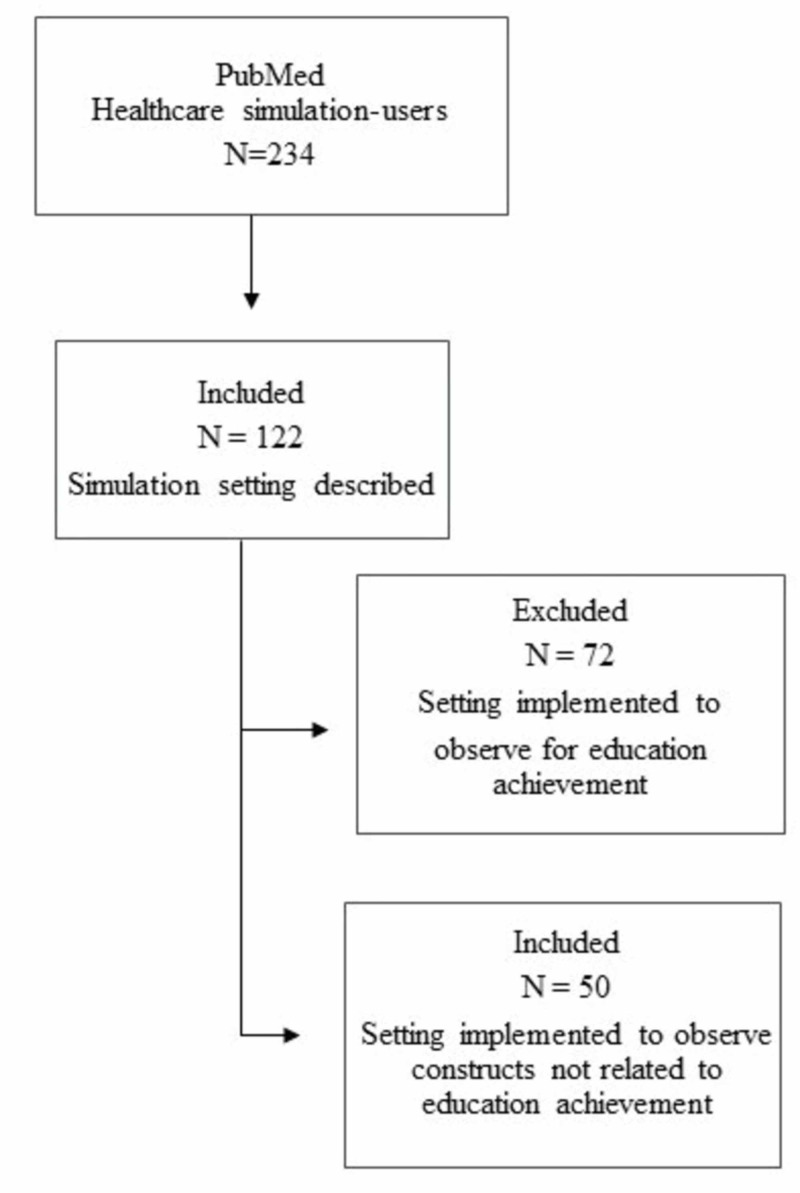
Article selection method

PubMed produced 234 HPE simulation-user articles. Of these 234 articles, 107 did not fit the description of a simulation setting framework and were excluded. The remaining 122 articles were screened and separated into two groups: studies that implemented a simulation setting for assessment in education achievement and studies that implemented simulation to assess constructs other than education achievement (e.g., training, process development). Of these, 72 articles related to reliability in assessing education achievement were excluded, resulting in a corpus of 50 articles for the study (Figure 2).

Article demographics

**Figure 3:**
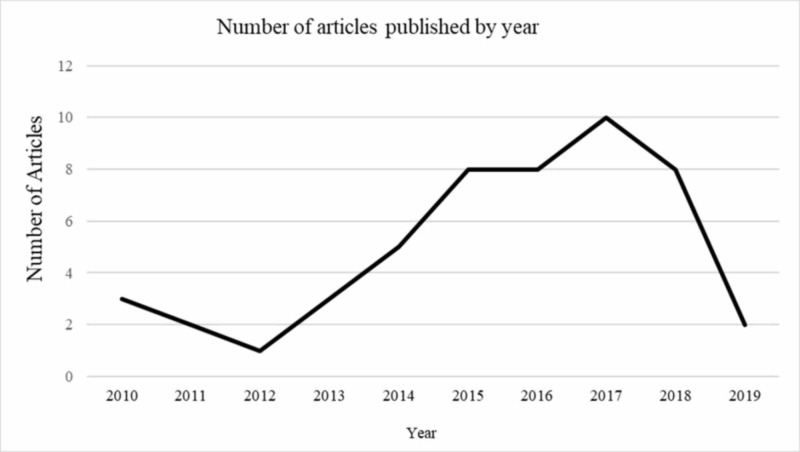
Articles published by year

Article research locations included North America (34%), Europe (20%), United Kingdom (16%), Asia (10%), unspecified (8%), Australia (6%), and South America (6%). The majority of the articles were published between 2015 and 2018 (Figure 3).

Sixty percent of the article did not specify the type of simulation environment. Simulation modality included task trainers (34%), simulated participants (32%), and articles that assessed simulator performance (50%) (Table 1).

**Table 1:** Demographic summary of setting attributes

Healthcare simulation setting attributes	Percentage of studies
ENVIRONMENT	
Unspecified	60%
In-house lab	16%
In-situ	12%
Multisite	6%
Non-hospital	4%
Portable	2%
MODALITY	
Task Trainer	34%
Simulated Patient	32%
Human Patient Simulator	12%
Animal Tissue	8%
Hybrid	6%
Augmented Reality	2%
Cadaver	2%
Inflatable Environmental Shell	2%
Unspecified	2%
CONSTRUCT ASSESSED	
Simulator Performance (i.e., task trainers, SPs)	50%
Subjective Interpretations	20%
Setting Dynamics	8%
Tool Performance	6%
Scenario Design	4%
Facilitator Performance	2%
Human Factors	2%
Process effectiveness	2%
Task Development	2%
Technical Skills	2%

Word frequency of reliability terms

**Figure 4:**
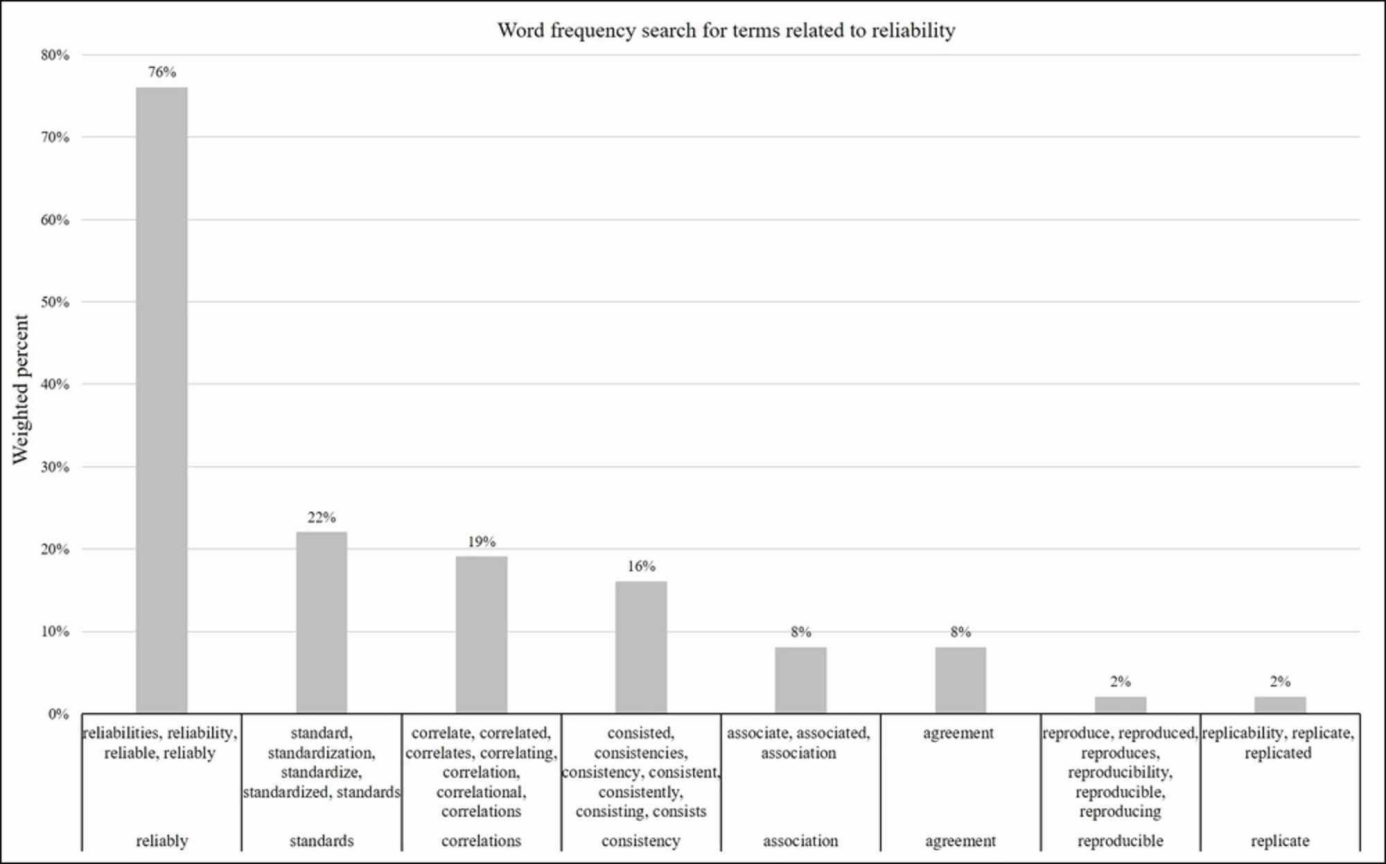
Word frequency search for terms related to reliability

Results from the word frequency search reveal eight words and their word-stems used across all articles from the PubMed search. Reliability terms include reliably (76%), standards (22%), correlations (19%), consistency (16%), agreement (8%), associations (8%), reproducible (2%), and replicate (2%) (Figure 4). These initiated a text-search query highlight each word across all articles.

Qualitative findings

**Figure 5:**
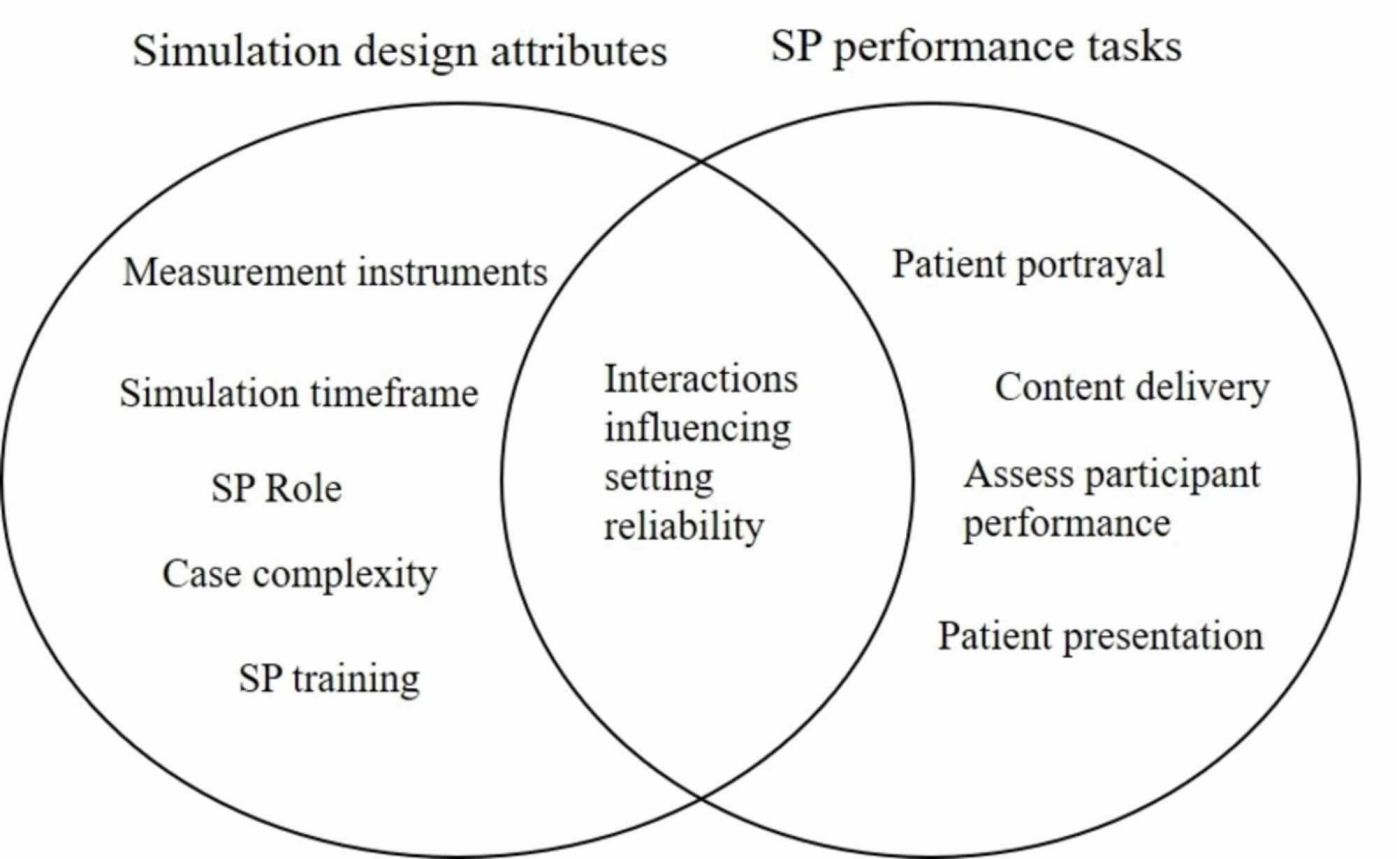
Constructs influencing simulation setting reliability

Of the 50 articles in the corpus, only six explicitly discuss simulation reliability (Table 2). Open and axial coding reveals that when combined, simulation design attributes (e.g., assessment tool items, timeframe, cases, SP performance training) and SP performance tasks (e.g., portrayal inaccuracies, content delivery, SP role), introduce errors that influence simulation setting reliability (Figure 5).

**Table 2:** Ontology of simulation setting reliability

Simulation setting reliability influenced by...
inefficiency to standardize SP performance during rehearsal
inconsistency and inaccuracy of SP performance and portrayal
inconsistency of SP rating performance ability using a measurement tool
inconsistency of SP performance, as a rater using a measurement tool, and patient portrayal

Drawing from these six articles, three SP training performance constructs (i.e., accuracy, consistency, standardization) including two subthemes for training SPs to achieve performance accuracy (i.e., reproducibility and replicability) and standardization (i.e., agreement and concordance) aim to mitigate errors. These constructs and related terms express how SPs are trained to target errors introduced by setting design attributes to achieve simulation setting reliability. In the following sections, presented are the attributes influencing simulation setting and SP performance achievement is presented, terms tied to the concept of reliability are discussed, and simulated participant training processes aimed at achieving setting reliability are examined.

Attributes influencing reliability

Simulation design attributes influencing SP performance included the complexity of SP task expectations (i.e., dual role performance as the patient and rater, use of a rating tool, and extended performance overtime) [25-27]. Also found were attributes that influence child SP performance (i.e., SP performance as a parent role, cognitive level of understanding the case purpose, and fatigue) [26]. Attributes influencing setting reliability include inefficiency in SP training, delivery of history content, physical presentation, patient portrayal (e.g., tone, demeanor), and inconsistent adherence to performance training protocols [25, 26, 28, 29].

Terms tied to the concept of simulation reliability

A frequent term surrounding the concept of reliability is consistency, specifically in relation to SP performance over time (n = 3 articles) [26, 29, 30]. MacLean et al. describe consistency as “SPs having a sound understanding of the purpose of the simulation and participant skills being targeted” [29]. In other words, setting reliability depends on how well SPs understand scenario objectives and the social cues needed to elicit participant behavior. To achieve setting reliability, SPs should consistently perform the designed activity requirements so that all participants face the same test conditions [25]. The idea of providing cues is to help navigate or clarify any gaps in the fidelity of the setting [31]. However, interactions mean that the effect of one factor depends on the level of the other factors potentially creating errors that influence setting reliability subsequently influencing participant performance activity goals.

Different terms are used when training SPs to achieve a specific performance construct (Table 3). Terms related to the achievement of SP performance standardization include calibration, agreement, and concordance (n = 2 articles) [25-27]. Another term identified was accuracy (n = 4 articles) [28-31]. Accuracy, in contrast, is represented through SP performance observations collected during simulation implementation. Accuracy in performance is measured by the “proportion of essential clinical features presented correctly in each encounter” [32]. As a measurement criterion, accuracy validates that SPs can consistently achieve performance reliability, thus “accuracy is another dimension of validity” [33].

**Table 3:** Reliability terms used by authors

Author	Reproducibility	Replicability	Consistency	Accuracy/Validity	Agreement	Concordance
Russell, 2011 [25]			X		X	
Russell, 2015 [26]			X		X	
Baig, 2014 [27]				X		X
Hill, 2013 [28]	X	X		X		
MacLean, 2018 [29]			X	X		
Shirazi, 2011 [30]			X	X		

Reproducibility and replicability are two unique terms (n = 1 article) that describe the accuracy of a particular type of performance. Although these terms may be considered synonymous, they have somewhat different meanings. Hill et al. define reproducibility as the “consistent performance of each SP across student interviews” and replicability as the “consistent performance of a number of SPs across each scenario” [28]. Based on the ontology of simulation design attributes believed to introduce setting errors, these lexical terms suggest how authors tailor training to achieve specific SP performance constructs.

Training approaches

An axial coding model adapted from Morrow, presents each author’s training process to achieve or assess SP performance. The model starts with the ontology of design attributes that introduce setting errors followed by phenomenological activities guiding the training approaches [34]. The following models examine how SPs are trained. The first three models examine the standardized training approaches for simulated patients to perform in a standardized fashion, thus transforming them into what is commonly known as a standardized patient. The models thereafter examine training standardized patients to achieve a specific performance construct. Simulated patients and standardized patients are defined by training experience [25, 26].

Training simulated patients for performance standardization using a standardized training approach

**Figure 6:**
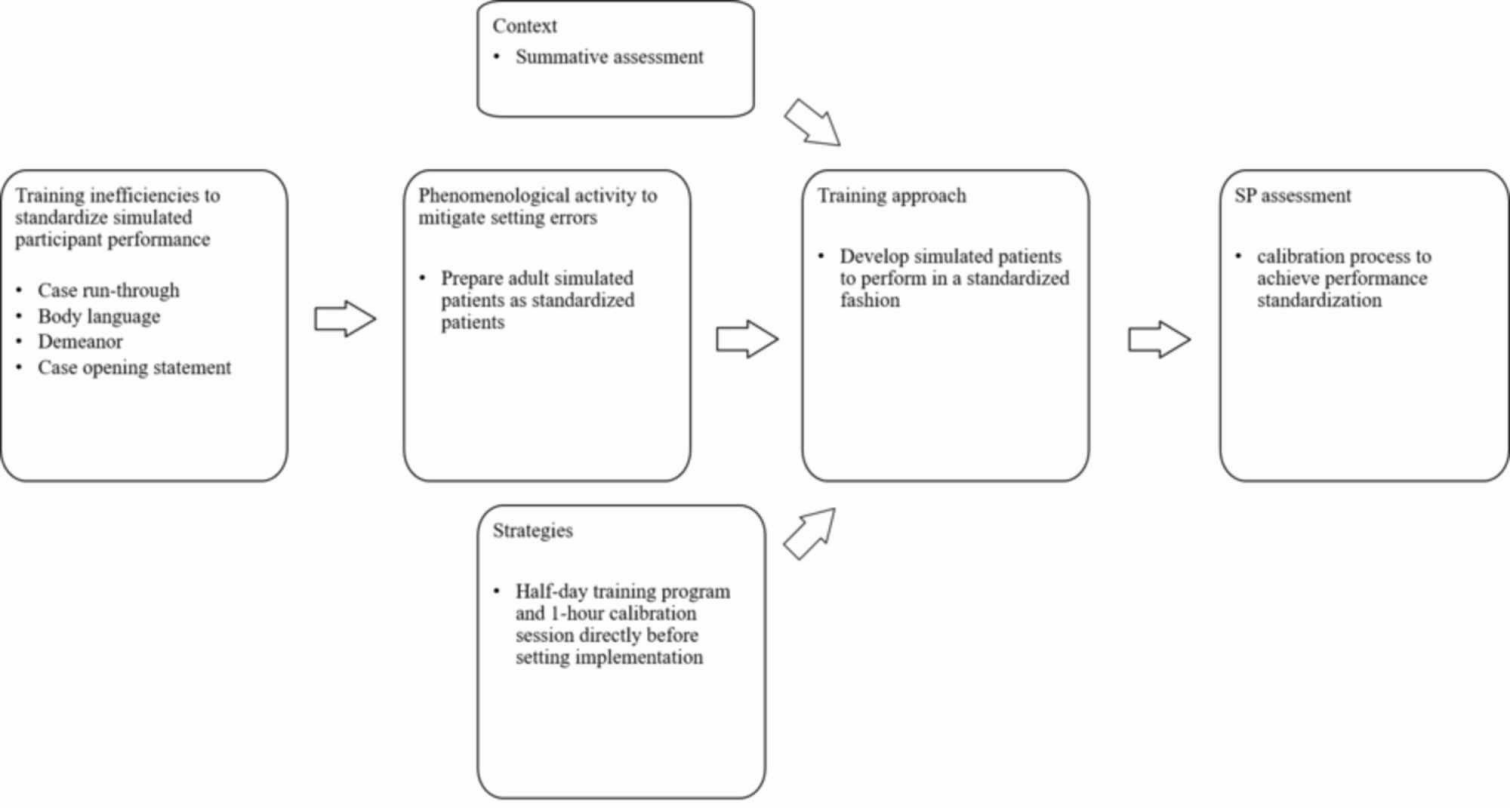
Training process to develop adult simulated patients to perform in a standardized fashion

To mitigate errors generated by SP performance (i.e., body language, demeanor, case opening statement), Russell et al. implement a one-hour calibration session directly before setting implementation (Figure 6) [25]. This calibration meeting is a strategic approach to standardize simulated patient performance [25]. A simulated patient “simply presents the symptoms and problems of an actual patient, whereas a standardize patient is trained to present these symptoms and problems in a consistent manner which does not vary from participant to participant or standardized patient to standardized patient” [25]. Put differently, simulated patients and standardized patients are defined by training experience.

During the calibration meeting, agreement must be reached by all SPs with a quality assurance observer. The agreement is made on expected performance skills during the delivery of the opening statement, demeanor, and cueing (what information to give, how and when) [25]. This meeting is “a short brieﬁng for role players held at the start of each day to remind SPs of expectations and also to follow up on any issues arising from the previous day(s)” [25]. The rationale for the strategic training approach is that participants will lead the interactions based on the preliminary questions asked to the standardized patient.

Training children simulated patients ​​​​​​for performance standardization

**Figure 7:**
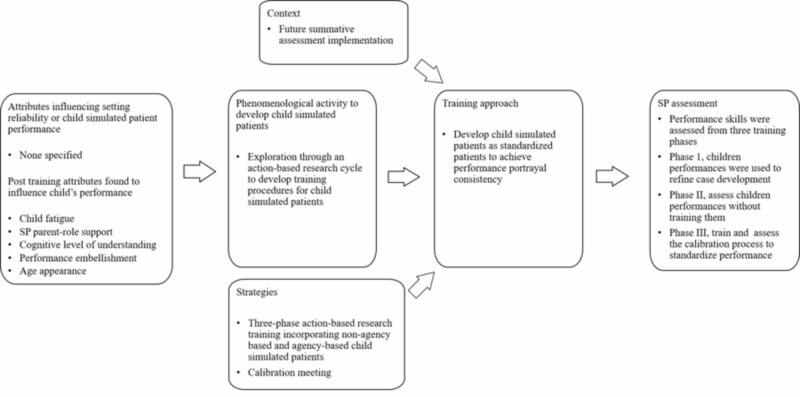
Training process to develop children simulated patients to perform in a standardized fashion

Using a three-phase action-based research process, the development to convert simulated child patients into standardized patients revealed that SP performance reliability was influenced by fatigue, SP parent role support, child’s cognitive level of understanding the purpose of the case, performance embellishment over time, and appearance of age (Figure 7) [26]. This study's plan was to implement children SPs into future learner summative assessments. Although quality assurance was observed, the study does not discuss simulation setting implementation, thus it is unclear if performance accuracy was achieved.

Training simulated patients for performance standardization and accuracy using a standardize training strategy

**Figure 8:**
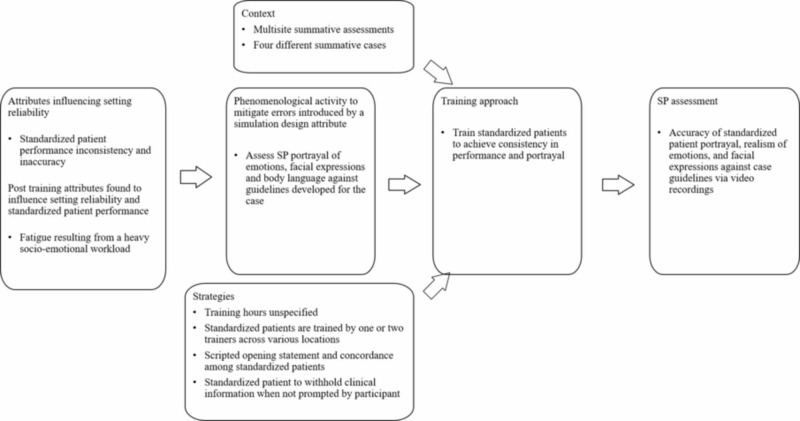
Training process to standardize patient performance accuracy

Simulation setting reliability is influenced by setting errors stemming from simulation design attributes and SP performance, specifically the inconsistency and accuracy between SP performance and portrayal (e.g., physical presentation, facial expressions, appearance, symptom representation) [28]. To mitigate these errors, Baig et al. train simulated patients to achieve performance accuracy by standardizing performance portrayal (Figure 8) [27]. Performance portrayal accuracy is achieved by “concordance between appearance, symptom representation, SP preparation and appropriateness of the case” [27]. This description correlates to the definition of consistency where SPs should have a sound understanding of the purpose of the simulation and skills assessed. Baig et al. maintain that concordance is how SPs achieve consistency [27]. This study assessed for SP performance consistency of emotions, facial expressions, and body language that were validated via performance observations informed by guidelines developed for each summative assessment case.

Training standardized patients for portrayal performance accuracy

**Figure 9:**
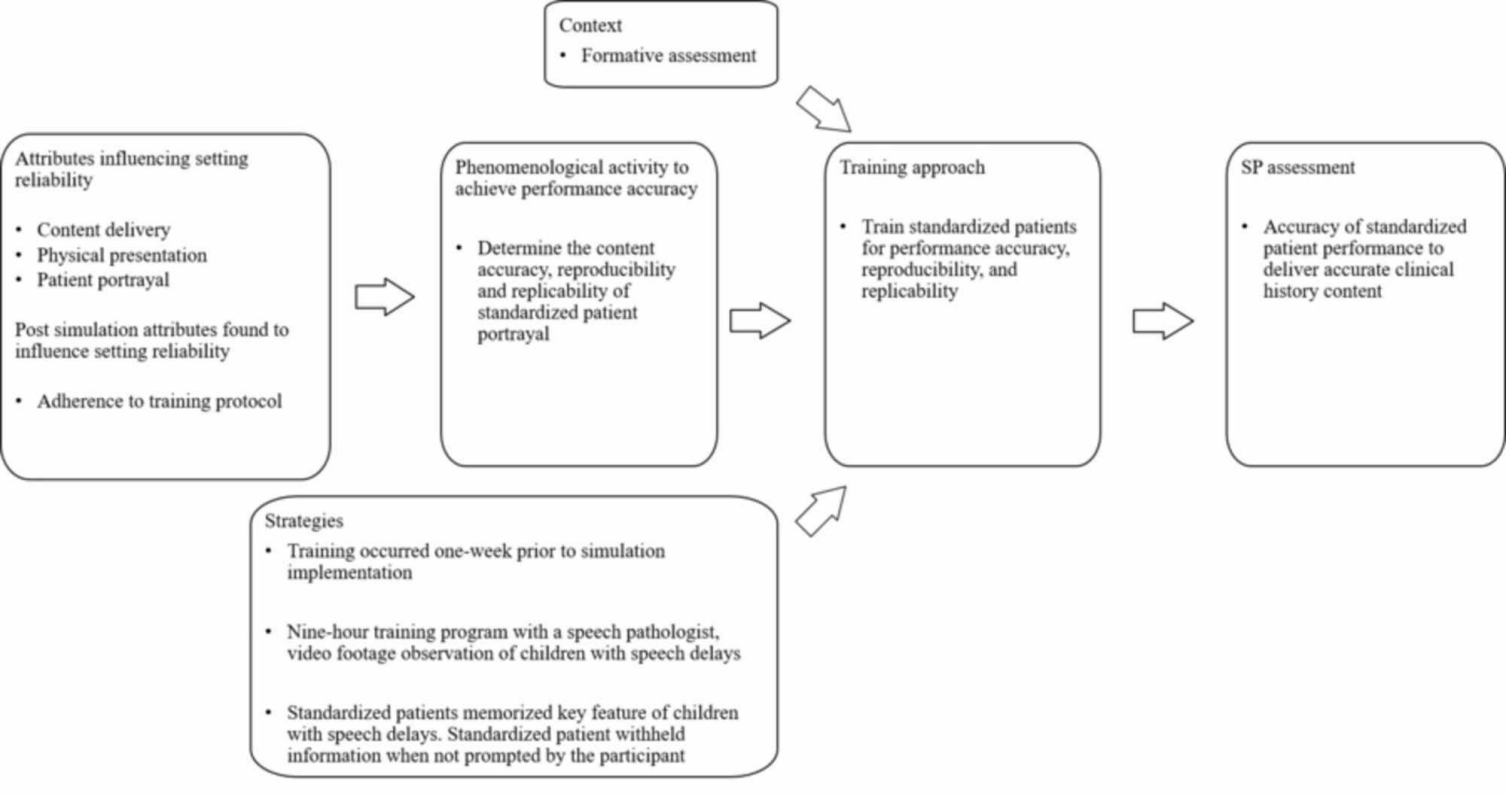
Training standardized patients for performance accuracy, reproducibility, and replicability

Standardized patients may construct performance errors in more than one way by content delivery, physical presentation and patient portrayal (Figure 9) [28]. Commonly, standardized patient performance training is sought to elicit participant behavior. In contrast, the purpose of this study was to train and assess SPs for accuracy in performance replicability, where a number of SPs perform in a consistent manner across each scenario, or performance reproducibility, where an individual SP presents the clinical scenario in the same way across each scenario [28]. In this study, accuracy is achieved by “the combination of SPs presenting all designated scenario features to students in the same way to a number of different students” [19]. The scenario features purposefully incorporated a heavy workload of socio-emotional performance.

In this study, SPs assumed a parent or grandparent role of a speech delayed child. The SP’s purpose was for participants to practice communication skills to acquire patient history. To achieve portrayal reproducibility across cases, SPs were trained by the same trainer using station-specific guidelines for the same case. Alternatively, other SPs recruited were trained to achieve portrayal replicability by using different SPs across each case. To achieve patient portrayal standardization, SPs were trained to withhold information if not specifically asked by participants. Standardized patients were trained to memorize key features of a speech delayed child, “the gold standard for SP portrayal” [33]. Post setting implementation revealed that fatigue from the heavy socio-emotional workload interfered with both setting and SP performance reliability.

Training standardized patients for performance consistency and accuracy in portrayal and assessment

**Figure 10:**
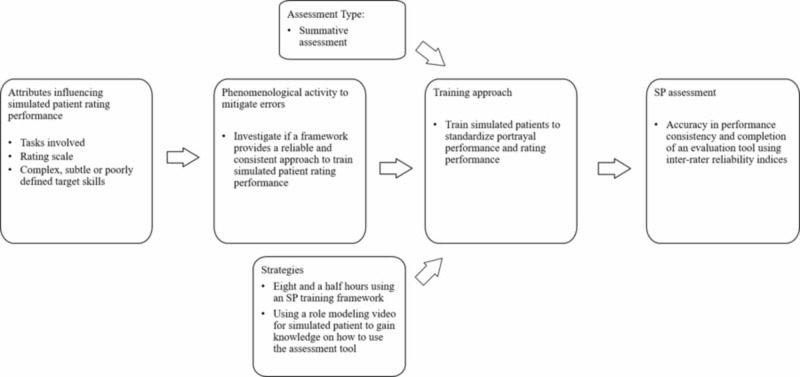
Training process to develop simulated patients as standardized performance raters

In this study, MacLean et al. trained simulated patients to perform as standardized patient assessors [29]. As raters, SPs were expected to complete performance-based evaluation instruments to assess participant communication skills. To mitigate performance error interfering with SP portrayal and errors introduced by design attributes (i.e., evaluation instrument), simulated patients were trained for eight and a half hours using a Nestel’s four-phase training framework (Figure 10) [35]. Different standardized patients were trained in the same case to achieve performance replicability. To achieve evaluative performance, simulated patients were trained to observe communication role-modeling behaviors using video recordings. Trainers evaluated SP performance activities and their ability to accurately and consistently complete evaluation instrument ratings over time. Through interrater reliability, SP’s use of the measurement instrument over time validated that SPs understood the purpose of the case and the participant skills being assessed. In other words, SPs were able to consistently complete the performance-based evaluation instrument.

Training unannounced simulated patients for performance consistency and accuracy in portrayal and assessment

In this study, simulated participants were resourced from a pool of nursing students. Shirazi et al. use the term ‘standardized patient’, however SPs were resourced from a pool of 25 nursing students that did not have any previous SP experience [30]. To remain consistent with role-players who do not have any experience in portraying SPs, the terminology, simulated participants as defined previously will apply to this particular discussion and axial coding model.

**Figure 11:**
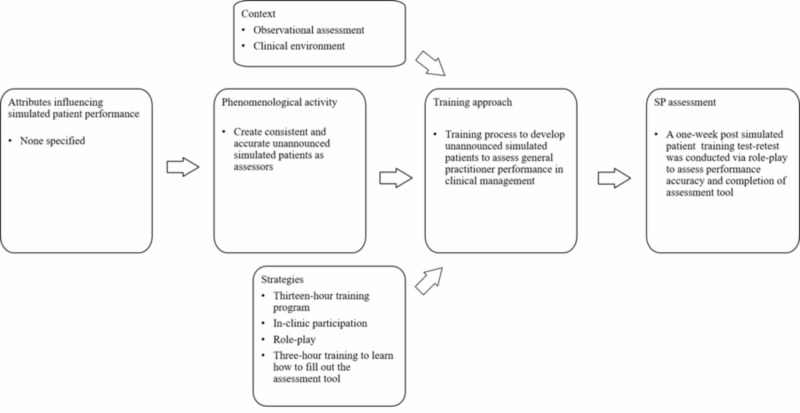
Training process to develop unannounced simulated patients to assess practitioner performance

This study aimed to develop SPs to achieve performance consistency and accuracy as an unannounced SP assessor (Figure 11). An unannounced SP plays a role in situations where clinicians are unaware that the SP person is not a real patient [20]. Simulated patients portrayed a depressive disorder combined with other illnesses (e.g., bipolar disorder, acute risk of suicide) and assessed a general practitioner’s management of the disorder. Different SPs were trained for a total of 13 hours on patient portrayal and completion of an assessment tool. Nursing students also participated in an ambulatory psychiatric clinic for training purposes. To assess SP performance and assessment consistency and accuracy, the authors performed a test-retest one week after training.

Discussion

When terminology used to describe reliability in healthcare simulations is combined as they are in this review, simulations are subject to variability in implementation due to their social aspects. From a social constructivist perspective, reliability in healthcare simulation is heavily dependent on an SP's consistency to achieve specific performance tasks that should provide the same stimuli to participants to achieve their goal-oriented activities. This finding is consistent with a study by Dieckmann et al., revealing that the experience of the simulation is influenced by the social characters of simulation, and the varying experiences of participants depend on how they interpret the same stimuli [17, 36-38]. Furthermore, when designing for simulation setting reliability, training approaches and strategies are not restricted to any type of design, therefore participant goal-oriented activities are vulnerable to errors stemming from interactions between simulation design attributes and SP performance tasks.

While this is not an emergent topic, clarifying these concepts and terminology is critical to effectively advance healthcare simulation education and assessment. The focus on the psychometric tools used in HPE does not consider the social aspect of simulation. Social practices add a layer of complexity that interferes with setting reliability that is supposed to provide participants fair and trustworthy conditions to achieve performance competency. To better understand the current nature of simulation setting reliability, further studies should be done to verify the replicability of the information acquired and explore the relationship between simulation design attributes underpinned by other learning paradigms (e.g., simulator equipment reliability employing a behaviorist paradigm). The research may provide insight into how to better achieve a fair and trustworthy simulation training environment by combining the different facets of simulation setting reliability that encompass simulation-based education and research.

Limitations

This review covers 2009-2019 and includes 50 articles from the 234 initially identified. Despite efforts to be as inclusive as possible, some articles may have inadvertently been missed. Additionally, the search used specific terminology and focused on specific databases, thus, this may have biased the inclusion of article samples. Searching a broader date range, database or terminology may have yielded additional articles.

## Conclusions

To achieve reliability in HPE and healthcare simulation, both domains seek to assess the consistency of a construct being measured. In HPE, reliability refers to the consistency of quality measures across a range of psychometric tests used to assess a participant’s medical aptitude. In healthcare simulation setting, reliability refers to the consistency of an SP performing a task that is tailored to mitigate errors introduced by simulation design attributes. Consequently, inconsistencies in SP performance subject participants to setting errors exposing them to unequal conditions that influence competency achievement.

## Appendices

Pubmed article search strategy

To reveal the initial results of healthcare simulation-users, copy and paste the full search string into PubMed.

(("Simulation Training"[Majr] OR “simulation design”[tiab] OR “simulation setting”[tiab] OR “simulation development”[tiab]) AND (Reliable[tiab] OR reliability[tiab]) AND ("2009/01/01"[PDat] : "3000/12/31"[PDat] ) AND English[lang]) NOT (Comment[sb] OR Case Reports[ptyp] OR Editorial[ptyp] OR Letter[ptyp] OR "Weightlessness Simulation"[Mesh] OR "Space Simulation"[Mesh] OR "Molecular Dynamics Simulation"[Mesh] OR "Molecular Docking Simulation"[Mesh] OR "Computer Simulation"[Mesh] OR "Malingering"[Mesh])
